# Epidural Analgesia during Open Radical Prostatectomy Does Not Improve Long-Term Cancer-Related Outcome: A Retrospective Study in Patients with Advanced Prostate Cancer

**DOI:** 10.1371/journal.pone.0072873

**Published:** 2013-08-19

**Authors:** Patrick Y. Wuethrich, George N. Thalmann, Urs E. Studer, Fiona C. Burkhard

**Affiliations:** 1 University Department of Anaesthesiology and Pain Therapy, University Hospital, Berne, Berne, Switzerland; 2 Department of Urology, University Hospital, Berne, Berne, Switzerland; The James Cook University Hospital, United Kingdom

## Abstract

**Background:**

A beneficial effect of regional anesthesia on cancer related outcome in various solid tumors has been proposed. The data on prostate cancer is conflicting and reports on long-term cancer specific survival are lacking.

**Methods:**

In a retrospective, single-center study, outcomes of 148 consecutive patients with locally advanced prostate cancer pT3/4 who underwent retropubic radical prostatectomy (RRP) with general anesthesia combined with intra- and postoperative epidural analgesia (n=67) or with postoperative ketorolac-morphine analgesia (n=81) were reviewed. The median observation time was 14.00 years (range 10.87-17.75 yrs). Biochemical recurrence (BCR)-free, local and distant recurrence-free, cancer-specific, and overall survival were estimated using the Kaplan-Meier technique. Multivariate Cox proportional-hazards regression models were used to analyze clinicopathologic variables associated with disease progression and death.

**Results:**

The survival estimates for BCR-free, local and distant recurrence-free, cancer-specific survival and overall survival did not differ between the two groups (*P*=0.64, *P*=0.75, *P*=0.18, *P*=0.32 and *P*=0.07). For both groups, higher preoperative PSA (hazard ratio (HR) 1.02, 95% confidence interval (CI) 1.01-1.02, *P*<0.0001), increased specimen Gleason score (HR 1.24, 95% CI 1.06-1.46, *P*=0.007) and positive nodal status (HR 1.66, 95% CI 1.03-2.67, *P*=0.04) were associated with higher risk of BCR. Increased specimen Gleason score predicted death from prostate cancer (HR 2.46, 95% CI 1.65-3.68, *P*<0.0001).

**Conclusions:**

General anaesthesia combined with epidural analgesia did not reduce the risk of cancer progression or improve survival after RRP for prostate cancer in this group of patients at high risk for disease progression with a median observation time of 14.00 yrs.

## Introduction

Interest has arisen on the potential effect of the anaesthetic technique applied during surgery for cancer on oncological outcome. This hypothesis is intriguing and based on the fact that not only the surgery itself but also the anaesthetic technique and the drugs applied (especially opioids) may affect the hosts’ immune response and consequently influence oncological outcome after surgery [1]. Results of recent studies addressing the potential impact of the epidural analgesia or anaesthetic technique applied during major oncological surgery on disease-specific outcome are ambivalent, either showing no difference, a reduced risk of recurrence or metastases during the initial follow up or improved survival for the first 1.5 years with no difference thereafter in favour of combined general anaesthesia with regional analgesia [2–4].

In prostate cancer 4 retrospective studies have been published. Biki et al. observed a positive effect of the thoracic epidural analgesia on biochemical recurrence (BCR)-free survival, however cancer–specific and overall survival were not assessed [5]. In a recent study from our institution we observed a significant effect of epidural analgesia on clinical progression-free survival but not on BCR-free, cancer-specific or overall survival [6]. Tsui et al. performed a secondary analysis in a patient population initially randomised to either general anaesthesia or combined anaesthesia to assess pain control. They were unable to detect a difference in BCR-free survival between the 2 groups [7]. Forget et al. found no association between peridural analgesia and BCR in more than 1000 patients with localized prostate cancer cT1-2 [8].

Based on the available literature the effect of anaesthesia technique on oncological outcome is most likely discrete. Prostate cancer, especially organ confined prostate cancer, generally has a relatively benign course of disease with excellent long term survival rates. To observe a difference a very long observation time is necessary.

In an attempt to circumvent these limitations, we chose a subgroup of patients who underwent a standardised surgical procedure for prostate cancer under general anaesthesia either combined with intra- and postoperative thoracic epidural analgesia (TEA) or with postoperative i.v. ketorolac and morphine for analgesia and were found to have locally advanced disease in the specimen. These patients were therefore at high risk for rapid disease progression and all had undergone surgery at least 10 years ago giving us the opportunity to evaluate long term outcome and survival.

**Figure 1:**
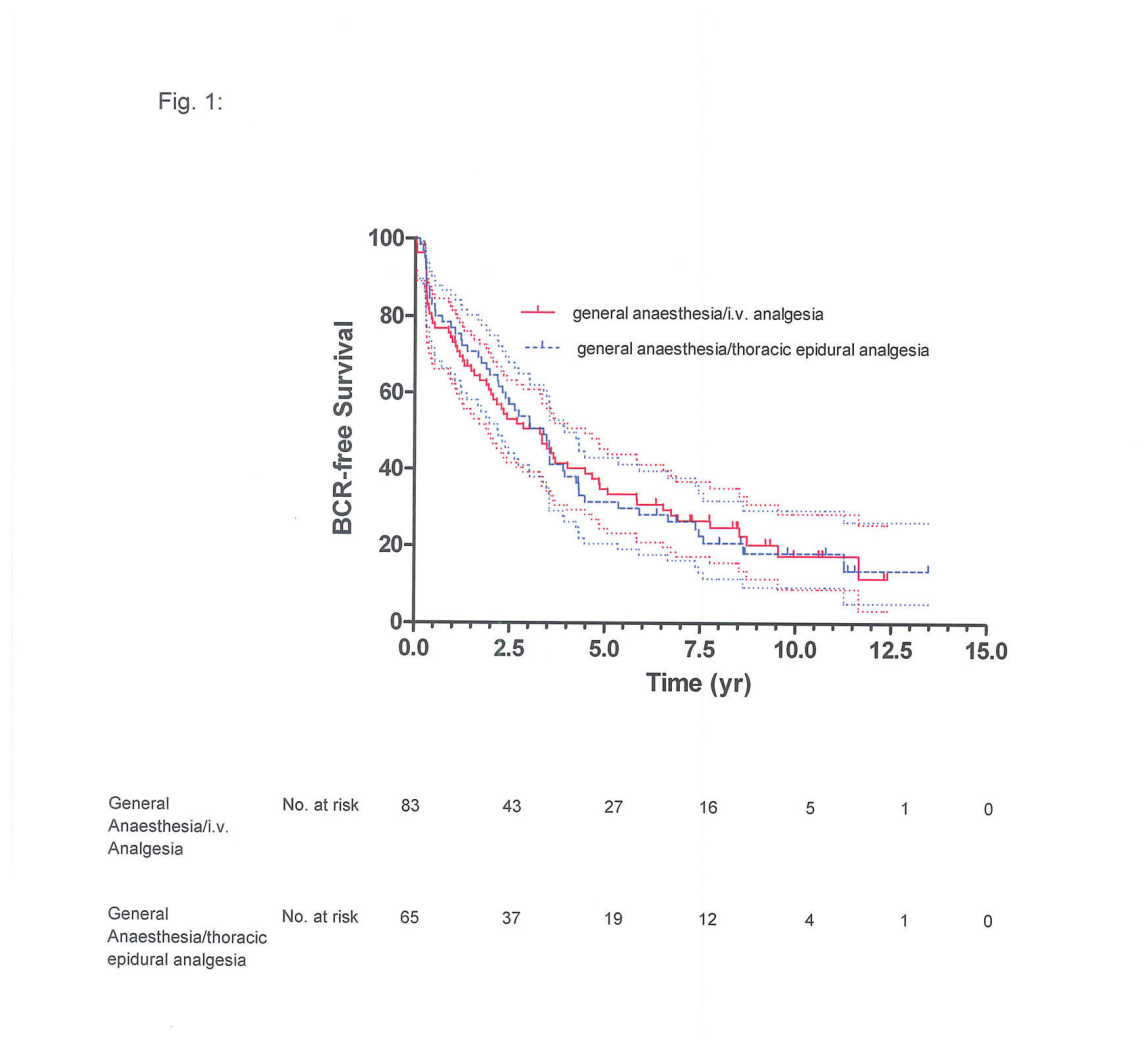
Biochemical recurrence-free survival of 81 patients given general anaesthesia with ketorolac-morphine analgesia and of 67 patients given combined general anaesthesia and thoracic epidural analgesia: Kaplan-Meier estimate with 95% confidence interval (upper and lower curves), with slash marks representing censored values (*P*=0.5225).

## Patients and Methods

The Institutional Review Board (Department Teaching and Research of the University Hospital, Bern) and Ethics Committee (Kantonale Ethik Kommission Bern, Switzerland) approved the retrospective review of the medical records of all patients who underwent open radical retropubic prostatectomy (RRP) in the Department of Urology of the University Hospital, Bern between January 1994 and December 2000 and waived the necessity for specific informed consent from each participant for this analysis.

**Figure 2:**
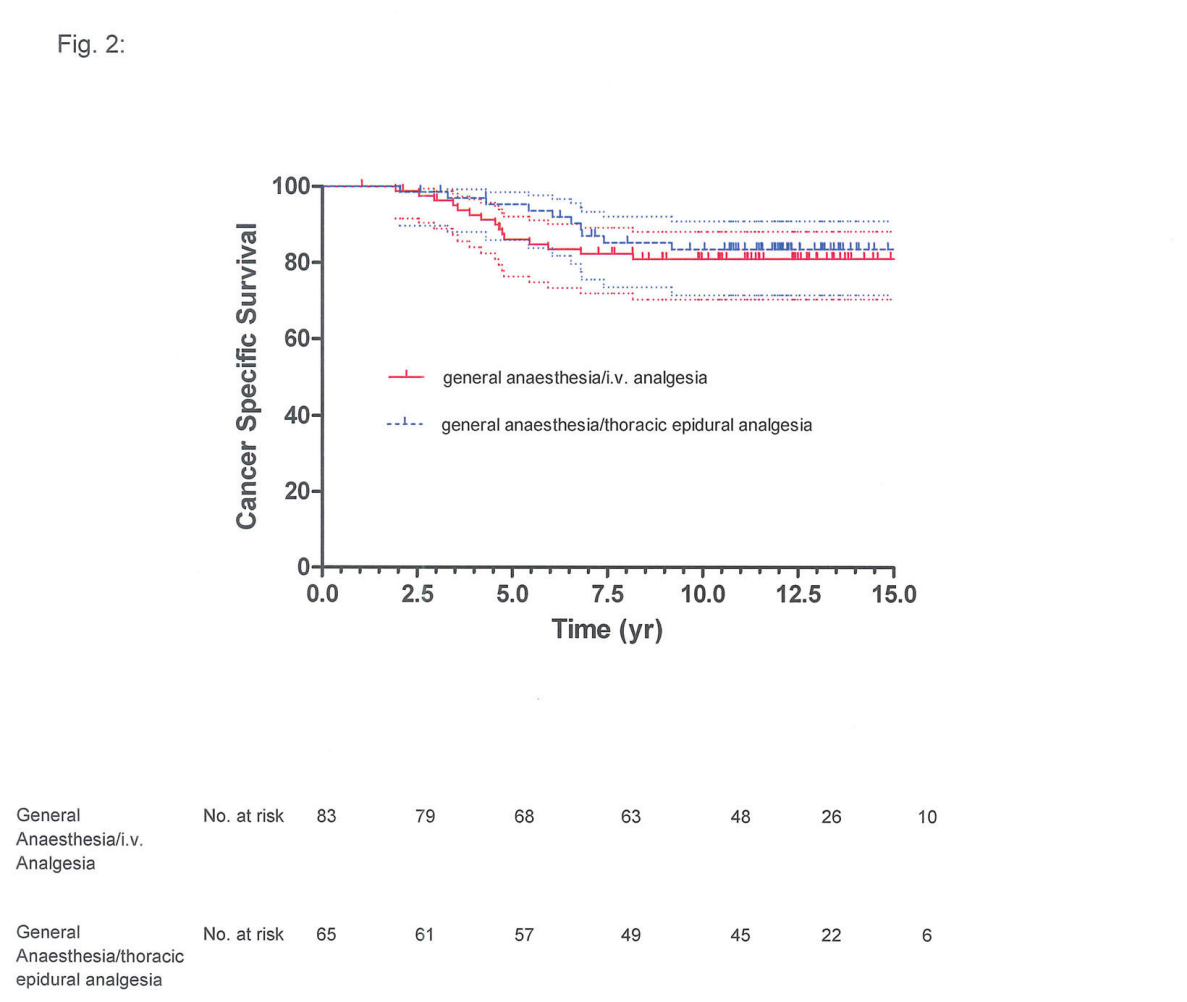
Prostate cancer-specific survival of 81 patients given general anaesthesia with ketorolac-morphine analgesia and of 67 patients given combined general anaesthesia and thoracic epidural analgesia: Kaplan-Meier estimate with 95% confidence interval (upper and lower curves), with slash marks representing censored (*P*=0.6834).

Only patients with pathological locally advanced prostate cancer stage pT3/4 who had undergone the same standardised RRP with pelvic lymph nodes dissection (PLND) were included in this analysis [9–11]. Patients who received neoadjuvant or adjuvant radiotherapy or androgen deprivation treatment (ADT) were excluded. ADT or radiotherapy was only delivered in case of clinical progression or a short PSA doubling time (< 9 mos).

All patients underwent the same general anaesthesia, including induction with thiopenthal (2-3 mg/kg), fentanyl (2 µg/kg), rocuronium (0.1 mg/kg) or atracurium (0.5 mg/kg). Anaesthesia was maintained with nitrous oxide and isoflurane. The patients were divided into two groups: patients who underwent combined general anaesthesia with intra- and postoperative thoracic epidural analgesia (TEA) (n=67) and patients given general anaesthesia alone with postoperative i.v. ketorolac and morphine for analgesia (n=81).

**Table 1:** Baseline characteristics.

	**General Anaesthesia and iv Analgesia (n=81)**	**General Anaesthesia and TEA (n=67)**	***P* Value**
**Age (yrs)**	63.83 [59.12-67.48]	63.61 [57.61-68.17]	0.52*
**Preoperative PSA (ng/ml)**	13.7 [10-21.2]	17.2 [9.2-31]	0.12*
**Fentanyl (mg)**	0.7 [0.5-0.75]	0.5 [0.4-0.65]	0.06*
**ASA physical status**			
**I**	21 (26)	12 (18)	0.04ǂ
**II**	45 (56)	50 (75)	
**III**	15 (18)	5 (7)	
**pT3a**	37 (46%)	25 (37%)	0.30
**pT3b**	42 (52%)	37 (55%)	
**pT4**	2 (2%)	5 (8%)	
**Positive Nodal Status (pN)**	35 (43)	30 (45)	0.85ǂ
**Specimen Gleason score (categorical)**			
**<7**	30 (37)	36 (5)	0.13ǂ
**7**	29 (36)	18 (27)	
**>7**	22 (27)	13 (19)	
**Need for Blood Transfusion**	12 (15)	11 (16)	0.79ǂ
**Need for ADT for recurrence**	39 (48)	30 (45)	0.51ǂ
**Need for Radiotherapy for recurrence**	20 (25)	13 (19)	0.69ǂ
**Positive Surgical Margin**	46 (57)	41 (61)	0.59ǂ

Data reported as number (%) and median [first-third quartile]. After matching the TEA group was the same as before matching. *: P values obtained from Wilcoxon rank sum test. ǂ: P values obtained from chi-square test. ASA=American Society of Anesthesiologists; PSA=prostate-specific antigen

TEA was activated at the beginning of RRP with bupivacaine 0.25% at a rate of 8-10ml/h. Patients with TEA received no COX-inhibitors intraoperatively. For postoperative epidural analgesia a standard solution containing 0.1% bupivacaine combined with 2 µg/ml epinephrine and 2 µg/ml fentanyl was administered at a rate of 8 to 15 ml/h for at least 48 hours after surgery. In addition 1000mg paracetamol i.v. was given every 6 hours. No COX-inhibitors were administrated in the group general anaesthesia/TEA analgesia postoperatively. None of these patients required supplemental systemic administration of morphine.

Patients without TEA received fentanyl boluses of 1-2 µg/kg intraoperatively at the discretion of the anaesthesiologist. Standard postoperative analgesia consisted of 30 mg ketorolac i.v. eight hourly and 1000 mg paracetamol i.v. six hourly during 48 hours. The first dose of ketorolac was administered at the time of fascial closure. Morphine 2 mg i.v. was given on request to supplement analgesia if necessary.

Evaluated baseline characteristics were age, American Society of Anesthesiologists (ASA) physical status classification, perioperative blood transfusions, total intraoperative dose of fentanyl, preoperative PSA, specimen Gleason score, surgical margins status and nodal (pN) status (according to the TNM classification 2002).

The two groups were compared for potential baseline confounders using chi square test for categorical variables and Wilcoxon rank sum test for continuous variables. A PSA value ≥ 0.2ng/ml was considered as BCR. BCR-free survival was calculated from operation to BCR or death, local recurrence-free and distant recurrence-free survival from operation to local or distant clinical progression or death resp, cancer-specific survival from operation to death due to tumour, and overall survival from operation to death of any cause. Patients were censored at the time of the last urological follow-up or death not due to events of interest. The endpoints were calculated using the Kaplan-Meier method.

To take into account potential confounding effects, multivariate Cox regression of each endpoint on analgesia group and all baseline characteristics was performed. As a supportive analysis, the endpoints were also compared between analgesia groups using univariate Cox regression in patients matched by propensity score, which was defined as the probability of receiving general anaesthesia with TEA, predicted from all baseline variables and calculated for each patient using logistic regression. The significance level for all parameters was 0.05. Due to the exploratory nature, no correction for multiple testing was applied. Statistical analysis was performed in collaboration with the Institute of Mathematical Statistics and Actuarial Science of the University of Berne using SPSS software version 18.0 (SPSS Inc., Chicago, Illinois, USA), SAS version 9.2 (SAS Institute Inc., Cary, NC, USA) and R software version 2.4.1 (The R Foundation for Statistical Computing, Vienna, Austria).

## Results

One hundred forty eight consecutive patients (median age 64 yrs, range: 45-75 yrs) were finally included. There was no statistically significant difference between the general anaesthesia/TEA (n=67) and general anaesthesia/i.v. analgesia (n=81) groups regarding baseline parameters with the exception of ASA physical status (*P*=0.04) (Table 1). After matching by propensity score, no significant difference between the groups could be detected in 67 matched pairs. Median intravenous morphine administration in the general anaesthesia/i.v. analgesia was 10mg [range 6-25mg] during the first 48 h postoperative.

**Table 2:** Cox regression of BCR free survival.

	**Hazard Ratio (95%CI) for BCR or Death**	***P* Value**
***Multivariate analysis before matching***		
**Anaesthesia with TEA vs. Anaesthesia with iv Analgesia**	0.91 (0.62-1.34)	0.6414
**Age (yrs)**	1.01 (0.98-1.05)	0.4133
**ASA Classification**	1.00 (0.73-1.38)	0.9830
**Preoperative PSA (ng.ml^-1^)**	1.02 (1.01-1.02)	**<.0001**
**Lymph nodes**		
**Positive vs. Negative**	1.66 (1.03-2.67)	**0.0372**
Specimen Gleason score*	1.24 (1.06-1.46)	**0.0073**
**Fentanyl (mg)**	1.67 (0.82-3.39)	0.1562
**Transfusion**		
**Yes vs. No**	0.68 (0.40-1.16)	0.1540
**Surgical margin**		
**Positive vs. Negative**	1.25 (0.84-1.86)	0.2734
***Univariate analysis after matching***		
**Anaesthesia with TEA vs. Anaesthesia with iv Analgesia**	1.00 (0.69-1.47)	0.9851

The median observation time was 14.0 yrs (range 10.9-17.8 yrs). After a median follow up of 11.3 yrs (range 1.2-16.5 yrs), 45/148 (30%) patients had died: 22/67 (33%) in the general anaesthesia/TEA group and 23/81 (28%) in the general anaesthesia/i.v. analgesia group (*P*=0.54). The cause of death was prostate cancer related in 32/148 (22%) patients: 14/67 (21%) general anaesthesia/TEA group and 18/81 (22%) in the general anaesthesia/i.v. analgesia group (*P*=0.68). In 13/148 (9%) patients death was not related to prostate cancer.

**Table 3:** Cox regression for local recurrence-free survival.

	**Hazard Ratio (95%CI) for BCR or Death**	***P* Value**
***Multivariate analysis before matching***		
**Anaesthesia with TEA vs. Anaesthesia with iv Analgesia**	1.19 (0.41-3.43)	0.7515
**Age (yrs)**	0.98 (0.90-1.07)	0.6441
**ASA Classification**	1.64 (0.71-3.76)	0.2466
**Preoperative PSA (ng.ml^-1^)**	0.98 (0.94-1.01)	0.2024
**Lymph nodes**		
**Positive vs. Negative**	1.12 (0.33-3.74)	0.8592
Specimen Gleason score*	1.33 (0.87-2.03)	0.1913
**Fentanyl (mg)**	1.91 (0.31-11.80)	0.4849
**Transfusion**		
**Yes vs. No**	1.10 (0.30-4.04)	0.8851
**Surgical margin**		
**Positive vs. Negative**	1.49 (0.48-4.64)	0.4887
***Univariate analysis after matching***		
**Anaesthesia with TEA vs. Anaesthesia with iv Analgesia**	1.16 (0.41-3.29)	0.7740

**Figure 3:**
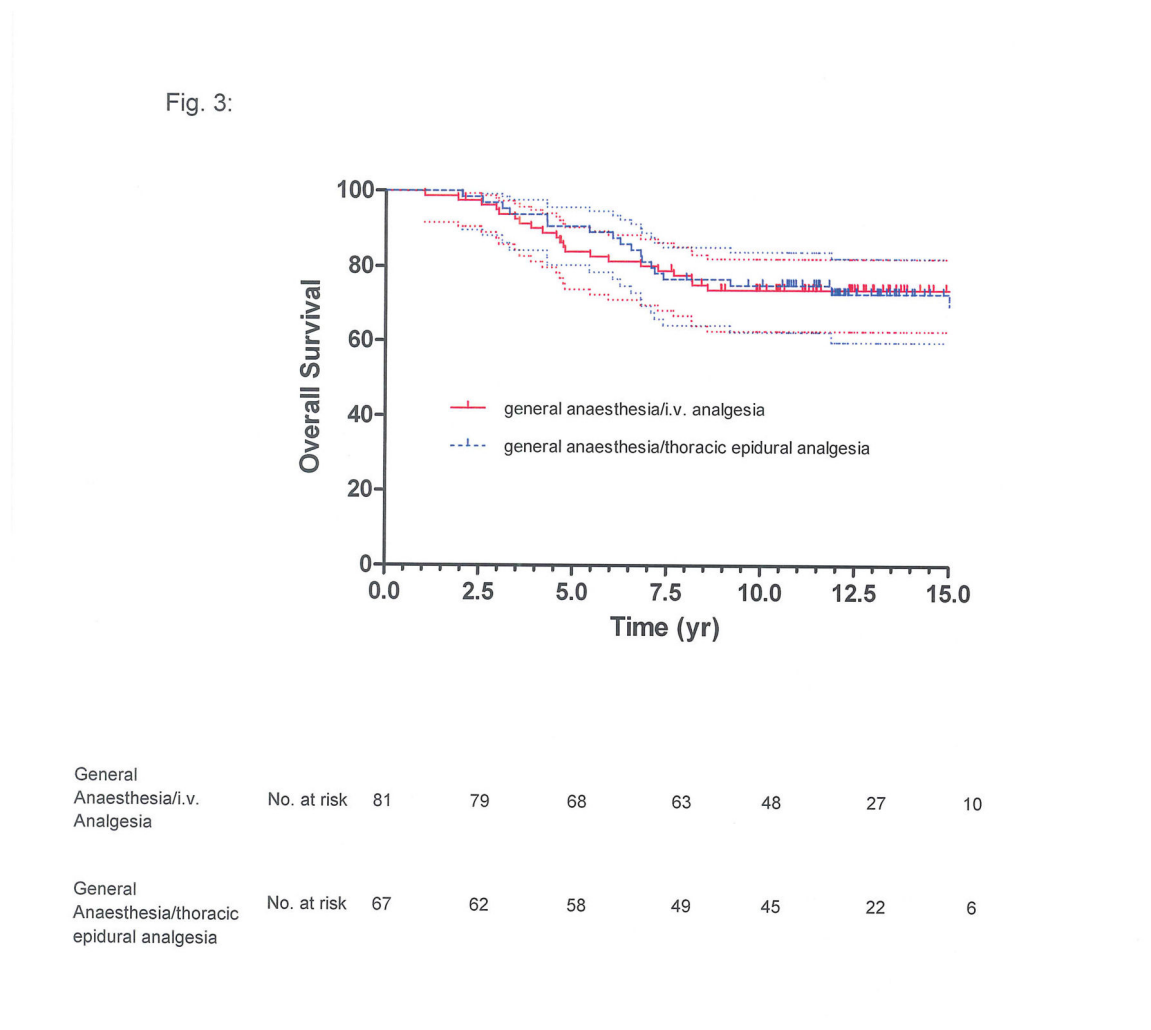
Overall survival of 81 patients given general anaesthesia with ketorolac-morphine analgesia and of 67 patients given combined general anaesthesia and thoracic epidural analgesia: Kaplan-Meier estimate with 95% confidence interval (upper and lower curves), with slash marks representing censored values (*P*=0.7459).

BCR-free survival at 5 years was 33% (95% confidence interval (CI): 22%-44%) for the general anaesthesia/TEA group and 32% (95% CI: 22%-43%) for the general anaesthesia/i.v. analgesia group, at 10 years it was 18% (95% CI: 10%-28%) and 21% (95% CI: 12%-30%), respectively (Figure 1). The respective cancer-specific survival was 89% (95% CI: 78%-95%) and 88% (95% CI: 79%-94%) at 5 years and 79% (95% CI: 66%-87%) and 78% (95% CI: 67%-86%) at 10 years (Figure 2); overall survival at 5 years was 82% (95% CI: 71%-89%) for the general anaesthesia/TEA group and 85% (95% CI: 75%-91%) for the general anaesthesia/i.v. analgesia group, at 10 years it was 69% (95% CI: 56%-78%) and 71% (95% CI: 60%-80%), respectively (Figure 3).

**Table 4:** Cox regression for distant recurrence-free survival.

	**Hazard Ratio (95%CI)** **for distant recurrence**	***P* Value**
***Multivariate analysis before matching***		
**Anaesthesia with TEA vs. Anaesthesia with iv Analgesia**	0.58 (0.27-1.29)	0.1816
**Age (year)**	0.98 (0.92-1.04)	0.4979
ASA*	0.96 (0.55-1.69)	0.8886
**Preoperative PSA (ng/ml)**	1.00 (0.99-1.01)	0.7711
**Lymph nodes**		
**Positive vs. Negative**	3.45 (1.25-9.53)	**0.0169**
Specimen Gleason score*	1.41 (1.00-1.98)	**0.0485**
**Fentanyl (mg)**	1.03 (0.24-4.45)	0.9670
**Transfusion**		
**Yes vs. No**	1.25 (0.47-3.32)	0.6611
**Surgical margin**		
**Positive vs. Negative**	1.15 (0.50-2.65)	0.7481
***Univariate analysis after matching***		
**Anaesthesia with TEA vs. Anaesthesia with iv Analgesia**	0.56 (0.26-1.25)	0.1573

**Table 5:** Cox regression of cancer specific survival.

	**Hazard Ratio (95%CI)for Cancer Specific Death**	**p Value**
***Multivariateanalysisbeforematching***		
**Anaesthesia with TEA vs. Anaesthesia with iv Analgesia**	1.51 (0.70-3.42)	0.3198
**Age (yrs)**	0.96 (0.19-1.05)	0.4755
**ASA Classification**	0.91 (0.52-1.58)	0.7298
**Preoperative PSA (ng.ml^-1^)**	1.00 (0.99-1.01)	0.9477
**Lymph nodes**		
**Positive vs. Negative**	2.94 (0.97-8.98)	0.0560
Specimen Gleason Score*	2.46 (1.65-3.68)	**<0.0001**
**Fentanyl (mg)**	1.79 (0.43-7.53)	0.4265
**Transfusion**		
**Yes vs. No**	1.29 (0.43-3.91)	0.6526
**Surgical Margin**		
**Positive vs. Negative**	1.07 (0.41-2.81)	0.8874
***Univariateanalysisaftermatching***		
**Anaesthesia with TEA vs. Anaesthesia with iv Analgesia**	0.96 (0.45-2.05)	0.9248

The survival estimates for BCR-free survival, local recurrence-free survival, distant recurrence–free survival, cancer-specific survival and overall survival did not differ between the two groups in multivariate analysis in all patients or in univariate analysis in matched patients (Tables 2, 3, 4, 5, 6).

**Table 6:** Cox regression of overall survival.

	**Hazard Ratio (95%CI)for Any Death**	***P* Value**
***Multivariate analysis before matching***		
**Anaesthesia with TEA vs. Anaesthesia with iv Analgesia**	1.79 (0.95-3.39)	0.0710
**Age (yrs)**	1.01 (0.96-1.07)	0.6772
**ASA Classification**	0.99 (0.62-1.60)	0.9725
**Preoperative PSA (ng.ml^-1^)**	1.00 (0.99-1.01)	0.6116
**Lymph nodes**		
**Positive vs. Negative**	1.50 (0.70-3.22)	0.2993
Specimen Gleason score*	1.85 (1.38-2.48)	**<0.0001**
**Fentanyl (mg)**	1.31 (0.40-4.29)	0.6537
**Transfusion**		
**Yes vs. No**	1.01 (0.42-2.46)	0.9808
**Surgical margin**		
**Positive vs. Negative**	1.23 (0.60-2.50)	0.5694
***Univariate analysis after matching***		
**Anaesthesia with TEA vs. Anaesthesia with iv Analgesia**	1.17 (0.63-2.17)	0.6105

Associated with higher risk for BCR were preoperative PSA (HR 1.02, 95% CI: 1.01-1.02, *P*<0.0001), specimen Gleason score (HR 1.24, 95% CI: 1.06-1.46, *P*=0.007), positive nodal status (HR 1.66, 95% CI: 1.03-2.67, *P*=0.04) (Table 2). The specimen Gleason score was a significant negative predictor for distant recurrence-free survival (HR: 1.41, 95% CI: 1.00-1.98, *P*=0.04) (Table 4), for cancer-specific survival (HR 2.46, 95% CI: 1.65-3.68, *P*<0.0001) (Table 5) and for overall survival (HR 1.85, 95% CI: 1.38-2.48, *P*<0.0001) (Table 6). Positive lymph node status was a negative predictor for distant recurrence-free survival (HR: 3.45, 95% CI: 1.25-9.53, *P*=0.01).

## Discussion

We report that epidural analgesia combined with general anaesthesia for radical prostatectomy did not improve BCR-free survival, cancer recurrence and survival in patients with locally advanced prostate cancer pT3/4 after a median observation time of 14 years.

This contrasts with 2 recently published studies including our prior publication, reporting that combined neuroaxial analgesia and general anaesthesia may be associated with a reduced risk of recurrence in prostate cancer [5,6]. In line with our observation Tsui et al. found no difference between the groups for biochemical recurrence-free survival in a secondary analysis of patients randomised to either general anaesthesia alone or combined general anaesthesia/epidural analgesia to evaluate pain control, blood loss, and the need for blood transfusion [7].

In the first study on this subject published by Biki et al., a difference in BCR-free survival was reported with better outcome in the patients with combined general anaesthesia/epidural analgesia. Although a sign of disease persistence or recurrence BCR-free survival is of questionable interest to the patient as it does not translate into cancer specific survival [12,13]. Additional treatment, such as androgen deprivation or radiotherapy can influence BCR and are not mentioned. The study of Biki et al. has some further limitations: the anaesthetic regimen was determined by the anaesthetist and was neither randomised nor consecutive patient groups. Most importantly, oncologically relevant information is lacking such as pathological tumor stage and the surgical technique is not mentioned.

In the retrospective study by Forget et al., they suggest that epidural analgesia did not influence BCR, but the use of sufentanil increased the risk of BCR [8]. However, the population analysed was heterogeneous, with many different and overlapping anaesthetic regimen, a short follow-up (median 38 months) and included patients receiving adjuvant therapies. In addition, another limitation is the very small number of patients not receiving systemic administration of sufentanil. Moreover sufentanil was added in the epidural mixture and a systemic effect cannot be ruled out and no information is given if the epidural analgesia was activated intraoperatively.

In the previous study from our institution on two consecutive patients groups a significant difference in clinical progression free survival was observed in favour of combined anaesthesia. However, no difference was found in BCR-free, cancer specific or overall survival [6].

Prostate cancer is a relatively benign disease and cancer specific survival estimates in organ confined prostate cancer are >95% at 10 years [14]. Therefore, hypothetically evaluation of more aggressive disease would be necessary to observe a difference in cancer specific survival and OS, which are the most relevant factors for the patient. Patients with pathologically proven non organ confined disease are at high risk of rapid disease progression. Reported BCR and cancer specific survival rates at 10 and 15 years are approximately 40% and 60% and 63-90% and 25-79%, respectively [15,16]. Outcome in our patients is in line with the reported literature confirming the representativeness of our cohort. One of the strengths of our study cohort is the uniform treatment: Surgery and PLND were performed in a standardized technique and none of the patients received adjuvant hormonal treatment or radiotherapy unless their PSA doubling time was <1 year or metastases were proven.

This may at least in part explain the difference seen in clinical progression free survival in this and the previous study. Patients with a rapid PSA recurrence are more likely to receive additional therapy based on their PSA which may interfere with the clinical course or explain the difference to the previous study also including patients with organ confined disease. ADT was not our endpoint. In a previous study on breast cancer a similar observation was made with a decrease in progression with combined anaesthesia [3].

The basis behind these observations is the potential effect of drugs on cancer outcome. Intraoperatively administrated fentanyl is purported to have a dose dependent effect on immune suppression [17]. Although in our study more fentanyl was given to patients not receiving epidural analgesia, we could not demonstrate a negative effect of the fentanyl dosage on survival.

Ketorolac, by contrast, may reduce cancer progression based on the over-expression of the COX-2 enzyme in prostate cancer cells compared with normal or benign hypertrophied cells. It is also associated with increased angiogenesis and proliferation in the animal model. Mean levels of COX-2 mRNA were shown to be 3.4-fold higher in prostate cancer tissue compared with the paired benign tissue in an in-vivo study [18]. COX-2 inhibitors induce apoptosis in prostate cancer cell lines [19,20]. The chemotherapeutic agent that has been studied most extensively in cancer is celecoxib. In a murine breast cancer model, celecoxib was shown to inhibit morphine-induced stimulation of COX-2, angiogenesis, tumor growth, metastasis and to lower mortality without compromising analgesia [21]. Perioperative use of COX-2 inhibitors, therefore, may have reduced the risk of tumor metastasis [22] and counterbalanced the negative effect of fentanyl in our cohort. However, a recent study did not support the use of celecoxib for patient with high risk cancer patients [23].

Other drugs which may be of relevance were applied perioperatively in our patients. Thiopental and isoflurane were used to induce and maintain anesthesia. Both drugs have a suppressive effect on T-lymphocyte proliferation [24–28]. Thiopental significantly enhanced postoperative metastasis after excision of the primary lung tumor in rats [29].

The majority of the above mentioned findings were observed in in-vitro or in animal studies. Among the factors potentially explaining differences between in-vitro or animal models and clinical findings are the duration and dosage of drug application as well as drug metabolism.

The present comparative single-centre study of consecutive patient cohorts has limitations inherent to all retrospective studies: it is not randomized and a selection bias cannot be definitively ruled out. As no intergroup differences were detected, the number of patients may not have been adequate. The long-term observation time in conjunction with the advanced disease stage and the consistency of our standardized surgical and anaesthetic techniques, however, should render this study ideal for the detection of a difference in disease outcome due to different anaesthetic techniques. Alternatively the effect of anaesthesia/analgesia on cancer specific survival may be of limited clinical value if of any at all.

Large prospective randomised multicenter studies on gynaecological, lung and colon tumors are now registered online at ClinicalTrial.gov and will in the future help to further clarify whether and why epidural anaesthesia/analgesia has an effect on cancer-specific outcome in patients undergoing surgery for cancer. However, the time needed to recruit patients and achieve an adequate observation period for cancer related outcome implies that results will not be evaluable for at least another 5 years. In the meantime retrospective studies may help to gain further insight.

### Conclusions

The hypothesis that general anaesthesia with epidural analgesia reduces the risk of cancer progression and/or improves survival in patients undergoing RRP for high-risk and locally advanced prostate cancer could not be confirmed in this comparative single-center observational study despite a long-term median minimal observation time of 10 years.
